# circ-*Iqsec1* induces bone marrow-derived mesenchymal stem cell (BMSC) osteogenic differentiation through the miR-187-3p/*Satb2* signaling pathway

**DOI:** 10.1186/s13075-022-02964-x

**Published:** 2022-12-14

**Authors:** Lixia Fan, Kaiyun Yang, Ruixuan Yu, Houde Hui, Wenliang Wu

**Affiliations:** 1 Department of Anesthesiology, Qilu Hospital of Shandong University, 107 Wenhua West Road, Jinan city, 250012 Shandong China; 2 Institute of Stomatology, Shandong University, 107 Wenhua West Road, Jinan city, 250012 Shandong China; 3 Department of Orthopaedics, Qilu Hospital of Shandong University, Jinan city, 250012 China

**Keywords:** circ-*Iqsec1*, BMSCs, miR-187-3p, *Satb2*, Osteogenic differentiation

## Abstract

**Background:**

Bone marrow-derived mesenchymal stem cells (BMSCs) are general progenitor cells of osteoblasts and adipocytes and they are characterized as a fundamental mediator for bone formation. The current research studied the molecular mechanisms underlying circRNA-regulated BMSC osteogenic differentiation.

**Methods:**

Next-generation sequencing (NGS) was employed to study abnormal circRNA and mRNA expression in BMSCs before and after osteogenic differentiation induction. Bioinformatics analysis and luciferase reporting analysis were employed to confirm correlations among miRNA, circRNA, and mRNA. RT-qPCR, ALP staining, and alizarin red staining illustrated the osteogenic differentiation ability of BMSCs.

**Results:**

Data showed that circ-*Iqsec1* expression increased during BMSC osteogenic differentiation. circ-*Iqsec1* downregulation reduced BMSC osteogenic differentiation ability. The present investigation discovered that *Satb2* played a role during BMSC osteogenic differentiation. *Satb2* downregulation decreased BMSC osteogenic differentiation ability. Bioinformatics and luciferase data showed that miR-187-3p linked circ-*Iqsec1* and *Satb2*. miR-187-3p downregulation or *Satb2* overexpression restored the osteogenic differentiation capability of BMSCs post silencing circ-*Iqsec1* in in vivo and in vitro experiments. *Satb2* upregulation restored osteogenic differentiation capability of BMSCs post miR-187-3p overexpression.

**Conclusion:**

Taken together, our study found that circ-*Iqsec1* induced BMSC osteogenic differentiation through the miR-187-3p/*Satb2* signaling pathway.

## Background

Osteoporosis (OP) is a widespread metabolic bone trait identified by reduced bone mineral density and bone quality, resulting in increased risk regarding bone fracture. It has a hazard ratio of 1.45 and 1.70 [1, 2]. It is broadly recognized that a disproportionate balance between osteoblast-related bone formation and osteoclast-mediated bone absorption in the bone marrow microenvironment leads to OP pathogenesis [3–6]. Mesenchymal stem cells (MSCs) differentiate directly into osteoblasts and then deposit mineralized extracellular matrix. MSCs have been most broadly investigated and applied in transplantation and therapy, both in basic experiments and in clinical trials [7]. Among these cells, BMSCs belong to the class of adult stem cells with the abilities of self-renewal and multi-directional division potential, which makes them able to transform into adipocytes, chondrocytes, osteoblasts, and nerve cells under different induction conditions [8, 9]. Nevertheless, the regulatory mechanisms are not clear.

Circular RNAs (circRNAs) are newly discovered RNAs that can form closed continuous rings covalently [10]. circRNAs are more stable than linear RNAs because they lack a free end for RNA enzyme-mediated degradation [11]. Research revealed that circRNA can regulate the microenvironment during osteogenic differentiation [6, 12, 13]. Former investigations suggested that circ-0016624 might sponge miR-98 to regulate *BMP2* expression during postmenopausal OP [14]. circ-*CDR1as* regulates osteoblastic differentiation of periodontal ligament stem cells through miR-7/*SMAD/GDF5* and *p38 MAPK* signaling pathways [15]. circ-*AFF4* modulates osteogenic differentiation of BMSCs through *SMAD1/5* pathway activation via the miR-135a-5p/*Irisin/FNDC5* axis [16]. However, circRNA roles in regulating osteoblastic differentiation is unclear. Therefore, more research is needed to investigate the basic molecular mechanisms of the circRNA regulatory network with respect to bone regeneration.

The current study determined that circ-*Iqsec1* promoted the induction of osteogenic differentiation and that knockdown of circ-*Iqsec1* significantly suppressed osteogenesis (OS) in BMSCs. miR-187-3p expression decreased as differentiation proceeded. In addition, circ-*Iqsec1* induced BMSC osteogenic differentiation via the miR-187-3p/*Satb2* signaling pathway. These data have proposed novel functions of circ-*Iqsec1* during BMSC osteogenic differentiation and highlighted its potential application as a novel therapy target in bone formation-related traits.

## Methods

### BMSC preparation, culture, and identification

Tibiae and femurs from BALB/c mice were extracted under sterile conditions to expose the bone marrow cavity, which was rinsed with saline. We collected and centrifuged the bone marrow filtrate at 225 × *g* for 5 min. The supernatant was discarded, and we resuspended the cells in HyClone low glucose (LG)-DMEM at 1×10^6^ cells per 100 μL. We gradually added the cell suspension to mouse lymphocyte separation medium (Sigma-Aldrich) in a 1:1 (v:v) ratio and centrifuged it at 1000 × *g* for 20 min. A milky turbid mononuclear cell layer was obtained. The cells were resuspended in LG-DMEM without FBS at 1×10^6^ cells per 100 μL before centrifuging at 225 × *g* for 5 min. The cells that were pelleted were resuspended in LG-DMEM complete medium containing 10% FBS and incubated in 5% CO_2_-saturated humidity at 37°C. The culture medium was changed every 3 days. The cells were sub-cultured in a 1:3 ratio when the cell confluence achieved 80~90%. BMSCs were passaged 3–4 times and utilized for the next steps. Fluorescein isothiocyanate (FITC-F) or phycoerythrin (PE) was applied for phenotypic analyses. *CD44*, *CD54*, *CD31*, *CD29*, *CD90*, *integrin-β1*, and *vWF* marker expressions were detected. IgG-matched isotype served as the internal control for all antibodies.

### Cell transfection

Satb2 gene overexpression vectors were made by putting Satb2 cDNA into a pcDNA3.1 vector. The miR-187-3p mimic/inhibitor and siRNA against circ-Iqsec1 (si-circ-Iqsec1) were synthesized by Genepharma (Suzhou, China). Lipofectamine 2000 (Invitrogen) was utilized for cell transfection following protocols.

### Bioinformatics analysis

We predicted correlations among miRNA, mRNA, and circRNA applying the online website http://starbase.sysu.edu.cn/.

### Multilineage bone marrow stem cell (BMSC) differentiation

To characterize BMSC abilities for multilineage differentiation, we cultivated 3rd-passage mouse BMSCs under various differentiation conditions. For adipocyte differentiation, we cultivated BMSCs in adipogenic differentiation medium. After 2 weeks, we determined adipocyte differentiation using Oil Red O staining. For osteoblast differentiation, we cultivated BMSCs in osteogenic differentiation medium and then stained them with alizarin red post 3 weeks to monitor overall survival (OS).

### Strand-specific NGS RNA-seq library preparation

Total RNA from BMSCs induced for 0 and 21 days was obtained utilizing TRIzol reagent (Invitrogen, CA, USA). Our group utilized VAHTS with 3 μg RNA from each sample. RNA-seq (H/M/R) library prep kits from Illumina (Vazyme Biotech Co., Ltd, Nanjing, China) were used to remove ribosomal RNA. RNA types, such as mRNAs and ncRNAs, were retained. RNA was treated applying 40 U RNase R (Epicenter) at 37°C for 3 h, followed by TRIzol purification. An RNA-seq library was prepared through KAPA stranded RNA-seq library prep kits (Roche, Basel, Switzerland), which we employed for NGS (Illumina HiSeq 4000 at Aksomics, Inc., Shanghai, China).

### RNA isolation and real-time PCR

Total RNA was obtained using TRIzol reagent (Invitrogen), followed by cDNA synthesis applying TransScript All-in-One First-Strand cDNA Synthesis SuperMix (Transgen Biotech, Beijing, China). Polymerase chain reaction (PCR) was conducted utilizing a Bio-Rad PCR instrument (Bio-Rad, CA, USA) and 2× Taq PCR master mix (Solarbio, Beijing, China) following all protocols. We calculated fold changes using the 2^−ΔΔCt^ approach. PCR primers were as follows:

circ-*Iqsec1*: forward 5′-GGCCTAAATCTCTTCAAC-3′ and reverse 5′-GCCAGUCUCGCUGCUGG-3′; miR-187-3p: forward 5′-TCGTGTCTTGTGTTGCAGCC-3′ and reverse 5′-GTGCAGGGTCCGAGGT-3′; *RUNX2*: forward 5′-ACTACCAGCCACCGAGACCA-3′ and reverse 5′-ACTGCTTGCAGCCTTAAATGACTCT-3′; *OCN*: forward 5′-AGCCACCGAGACACCATGAGA-3′ and reverse 5′-GGCTGCACCTTTGCTGGACT-3′; *Satb2*: forward 5′-GCAGTTGGACGGCTCTCTT-3′ and reverse 5′-CACCTTCCCAGCTTGATTATTCC-3′; *U6*: forward 5′-CGCTTCGGCAGCACATATACTAAAATTGGAAC-3′ and reverse 5′-GCTTCACGAATTTGCGTGTCATCCTTGC-3′; and *GAPDH*: forward 5′-GGAGCGAGATCCCTCCAAAAT-3′ and reverse 5′-GGCTGTTGTCATACTTCTCATGG-3′.

### Dual-luciferase reporter assay

Our group amplified *Satb2* and circ-*Iqsec1* 3′-UTR and miR-187-3p binding sites through PCR. We inserted sequences into multiple cloning sites in the pMIR-REPORT luciferase miRNA expression reporter vector. We co-transfected HEK293T cells with 0.1-μg luciferase reporter vectors containing wild-type (WT) or mutant-type (MUT) *Satb2* or circ-*Iqsec1* 3′-UTR and miR-187-3p mimic or miR-control utilizing Lipofectamine 2000 (Invitrogen, CA, USA). We computed relative luciferase activity by normalizing firefly luminescence to Renilla luminescence through a dual-luciferase reporter assay system (Promega, WI, USA) following protocols two days post-transfection.

### Animals and cell transplantation

We infected BMSCs at the 4th passage utilizing lentivirus (si-circ-*Iqsec1*, miR-187-3p mimic, or *Satb2* overexpression vector) and cultured in osteogenic differentiation medium for 7 days prior to in vivo research. After the BMSCs were trypsinized and directly resuspended in DMEM, we cultured the BMSCs with SynthoGraft (β-tricalcium phosphate; Bicon) for 1 h at 37°C. They were then centrifuged at 150 × *g* for 5 min and implanted into 2 symmetrical sites in the dorsal subcutaneous space in six 6-week-old BALB/c nude mice. The ethical review committee of Qilu Hospital of Shandong University approved the animal experiments.

### Immunohistochemical analyses

We harvested specimens 8 weeks post-transplantation from mice euthanized by CO_2_ asphyxiation. We decalcified specimens in 10% EDTA (pH 7.4), which we dehydrated and embedded in paraffin. We cultured sections overnight at 4°C with primary antibodies against *OCN* and *RUNX2* and then for 1 h at 37°C with secondary antibodies (Abcam). We stained the sections using 3,3-diaminobenzidine. We counterstained with hematoxylin and examined under an Axiophot light microscope (Zeiss, Oberkochen, Germany).

### Statistical analyses

The continuous variables are represented as the mean ± SD. For comparisons, one-way variance of analysis was conducted through GraphPad Prism (GraphPad, CA, USA). *P* ≤ 0.05 informed statistical significance.

## Results

### BMSC characterization and differentiation

**Fig. 1:**
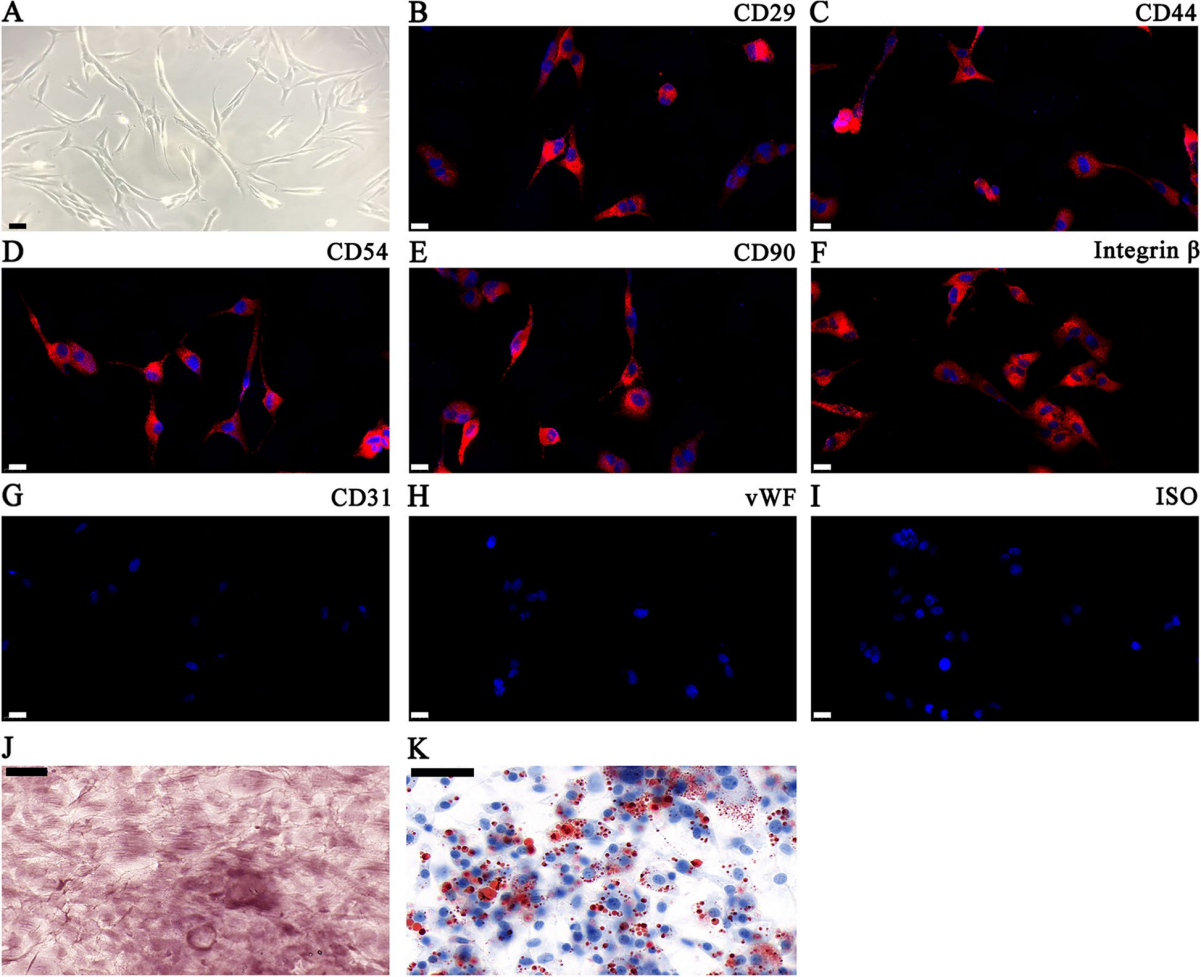
Isolation and identification of differentiation and classical phenotypes of BMSCs. **A** Phase-contrast images showing BMSC morphology. Scale bars: 20 μm. **B**–**I** Surface antigen expression in mouse BMSCs at the 3rd passage. Cells stained positive for mesenchymal stem cell markers *CD44*, *CD29*, *CD90*, *CD54*, and integrin-β. Endothelial markers *CD31* and *vWF* were used as negative markers. ISO was applied as control. Scale bars: 20 μm. **J**, **K** The BMSC differentiation potential was detected through alizarin red (**J**) and Oil Red O (**K**) staining. Scale bars: 50 μm

We made BMSCs from bone marrow obtained from BALB/c mice tibiae and femurs. The cultured BMSCs had a typical morphology with a spindle structure (Fig. 1A). They were positive for known MSC markers *CD44*, *CD90*, *CD54*, *CD29*, and *integrin-β1*, yet did not express endothelial cell markers *CD31* and *vWF* (Fig. 1B–I). The study also found that the isolated BMSCs had osteoblast and adipocyte differentiation ability, as demonstrated by Oil Red O (Fig. 1J) and alizarin red (Fig. 1K) staining.

### circ-Iqsec1 plays a role during BMSC osteogenic differentiation

**Fig. 2:**
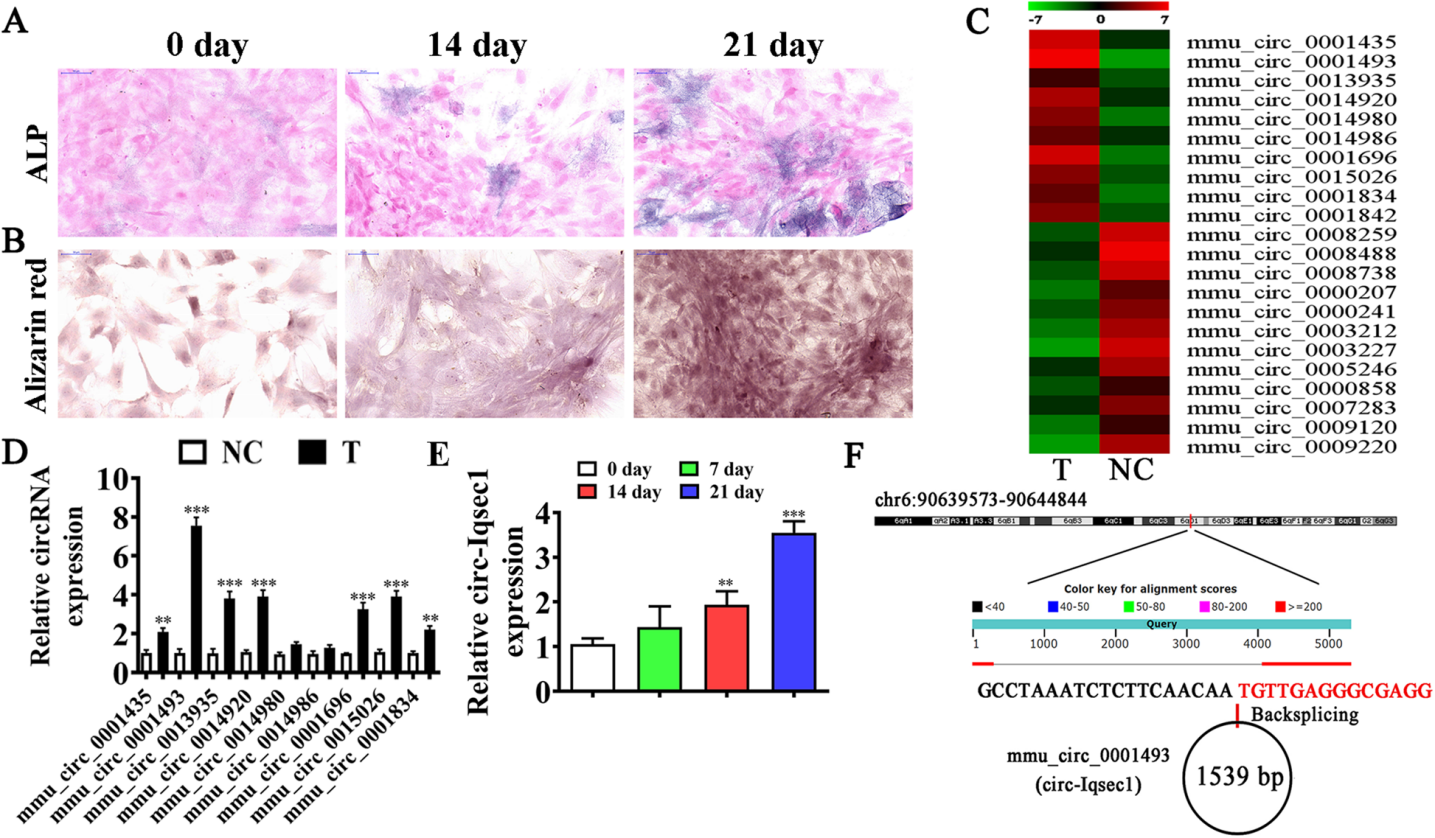
circ-*Iqsec1* plays a role during osteogenic BMSC differentiation. **A** *ALP* staining shows the BMSC osteogenic differentiation post osteogenic induction for 0, 2, and 3 weeks. **B** Alizarin red staining showing BMSC osteogenic differentiation post-osteogenic induction for 0, 2, and 3 weeks. **C** NGS was used for circRNA expression detection in BMSCs after osteogenic induction for 0 (NC) and 3 (T) weeks. **D** RT-qPCR detection showing abnormal circRNA expression. Results are denoted by the mean ± SD. ^**^P < 0.01, ^***^P < 0.001 vs. NC. **E** RT-qPCR data giving circ-Iqsec1 expression during osteogenic differentiation of BMSCs. Output is denoted as the mean ± SD. ^**^P < 0.01, ^***^P < 0.001 vs. 0 day. **F** Genomic loci of *Iqsec1* and mmu_circ_0001493

To identify the osteogenic differentiation ability, we induced BMSCs with an osteogenic differentiation induction medium for 0, 14, and 24 days. *ALP* staining (Fig. 2A) and alizarin red staining (Fig. 2B) showed that the osteogenic differentiation ability was time-dependent. High-throughput sequencing showed that osteogenic differentiation resulted in the abnormal expression of circRNA (Fig. 2C). RT-qPCR detection showed nine high-expression circRNAs based on the sequencing data. The data showed that only mmu_circ_0001493 expression increased significantly in the osteogenic differentiation-induced group (Fig. 2D). Furthermore, the RT-qPCR results illustrated that increased mmu_circ_0001493 expression in BMSCs depended on the induction time (Fig. 2E). This suggested that mmu_circ_0001493 functions in the osteogenic differentiation of BMSCs. mmu_circ_0001493 originated by cyclizing two exons from *Iqsec1*, located at chr6:90639573-90644844. *Iqsec1* was 5271 bp, and the spliced mature circRNA was 1539 bp (Fig. 2F). Therefore, mmu_circ_0001493 was also called circ-*Iqsec1* by our laboratory.

**Fig. 3:**
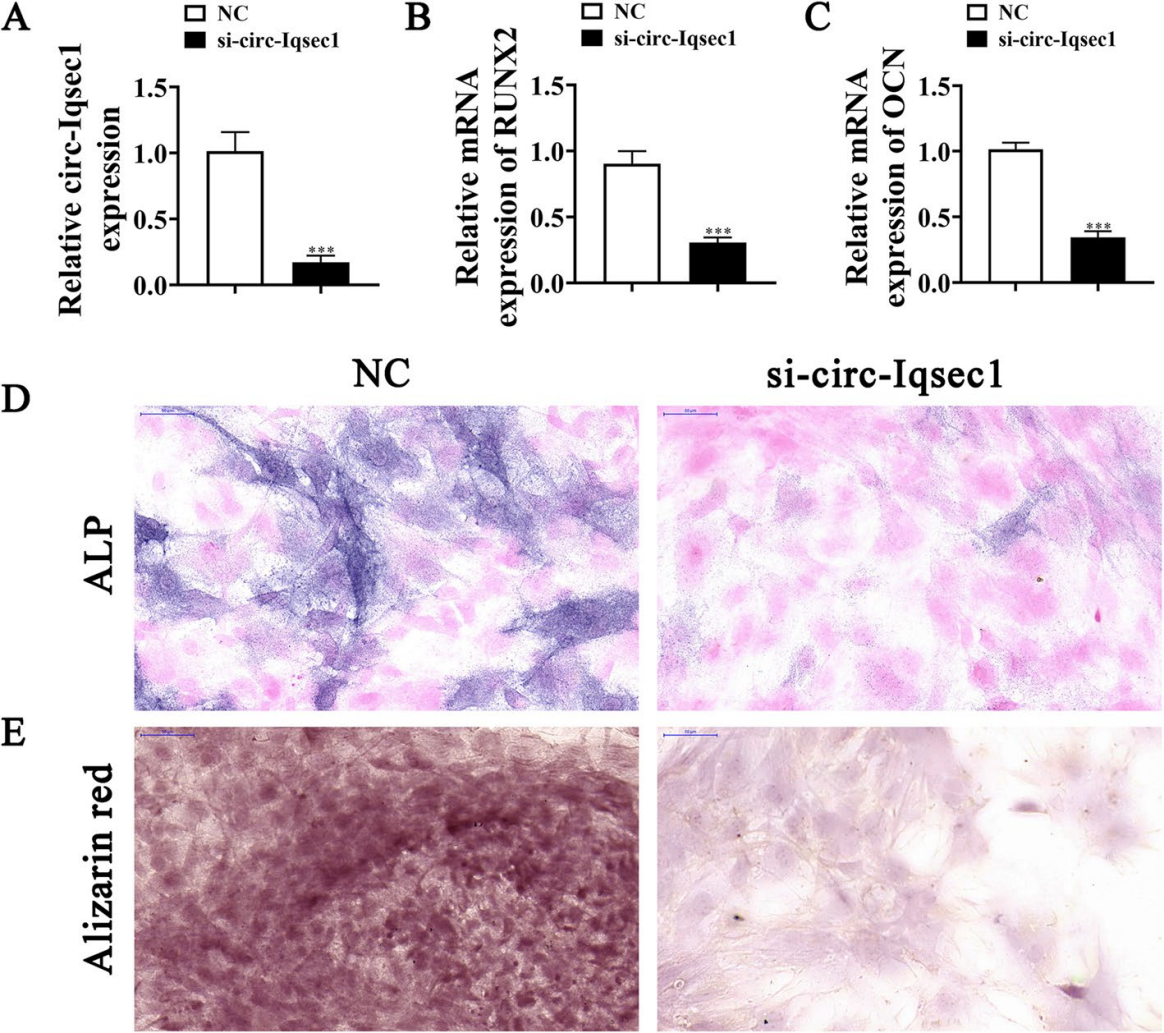
Downregulation of circ-*Iqsec1* significantly decreases BMSC osteogenic differentiation ability. **A** RT-qPCR data showing circ-Iqsec1 expression in BMSCs after siRNA transfection against circ-*Iqsec1* or NC. Outcomes are expressed as the mean ± SD. **B**, **C** RT-qPCR data showing *RUXN2* and *OCN* expression after osteogenic induction for 3 weeks. Outcomes are represented as the mean ± SD. **D**, **E** Immunohistochemical staining for *ALP* and alizarin red staining for calcium showing BMSC osteogenic differentiation ability post silencing circ-Iqsec1. ^***^P < 0.001 vs. NC

To further identify whether circ-*Iqsec1* could participate in BMSC osteogenic differentiation, siRNA against circ-Iqsec1 (si-circ-*Iqsec1*) was constructed and transfected into BMSCs. The data showed that circ-Iqsec1 expression decreased significantly post-silencing circ-*Iqsec1* (Fig. 3A). RT-qPCR data showed that circ-*Iqsec1* downregulation inhibited *RUNX2* (Fig. 3B) and *OCN* (Fig. 3C) expression. Immunohistochemical staining for *ALP* (Fig. 3C) and alizarin red staining for calcium (Fig. 3D) showed that circ-*Iqsec1* downregulation decreased BMSC osteogenic differentiation ability.

### Satb2 plays a role during osteogenic differentiation of BMSCs

**Fig. 4:**
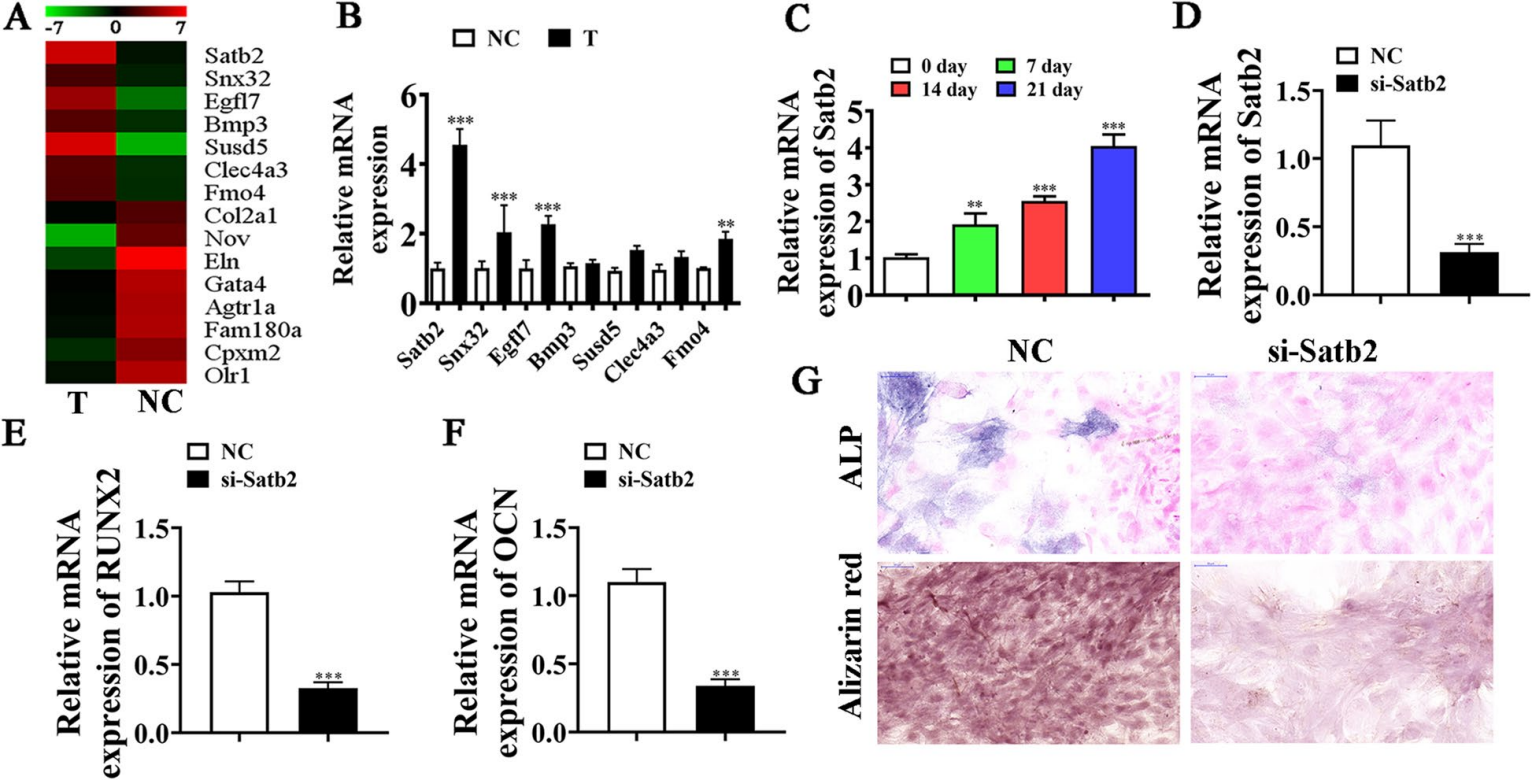
*Satb2* plays a role in osteogenic differentiation of BMSCs. **A** NGS was applied for mRNA expression detection in BMSCs after osteogenic induction for 0 (NC) and 3 (T) weeks. **B** RT-qPCR results showing abnormal mRNA expression. Data are denoted as the mean ± SD. ^**^P < 0.01, ^***^P < 0.001 vs. NC. **C** RT-qPCR detection giving *Satb2* expression during BMSC osteogenic differentiation. Data are denoted as the mean ± SD. ^**^P < 0.01, ^***^P < 0.001 vs. 0 day. **D** RT-qPCR detection giving *Satb2* expression in BMSCs after siRNA transfection against Satb2 (si-Satb2) or negative control (NC). Data are expressed as the mean ± SD. **E**, **F** RT-qPCR data giving *RUXN2* and *OCN* expression after osteogenic induction for 3 weeks. Outputs are expressed as the mean ± SD. **G** Immunohistochemical staining for *ALP* and alizarin red staining showing BMSC osteogenic differentiation ability post silencing Satb2. ^***^P < 0.001 vs. NC

High-throughput sequencing showed that osteogenic differentiation resulted in the abnormal expression of mRNA (Fig. 4A). RT-qPCR detection and sequencing data showed seven high-expression mRNAs: *Satb2*, *Snx32*, *Egfl7*, *Bmp3*, *Susd5*, *Clec4a3*, and *Fmo4*. However, only *Satb2* expression increased significantly in the osteogenic differentiation-induced group (Fig. 4B). RT-qPCR data further showed that *Satb2* expression increased in BMSCs in a time-dependent way following induction (Fig. 4C). This suggests that *Satb2* functioned in BMSC osteogenic differentiation.

To further identify whether *Satb2* participates in BMSC osteogenic differentiation, siRNA against *Satb2* (si-*Satb2*) was constructed and transfected into BMSCs. The result showed that *Satb2* expression decreased significantly after silencing of *Satb2* (Fig. 4D). RT-qPCR detection showed that downregulation of *Satb2* inhibited *RUNX2* (Fig. 4E) and *OCN* (Fig. 4F) expression. Immunohistochemical staining for *ALP* and alizarin red staining for calcium showed that *Satb2* downregulation reduced BMSC osteogenic differentiation ability (Fig. 4G). This suggested that Satb2 functioned during BMSC osteogenic differentiation.

### miR-187-3p links Satb2 and circ-Iqsec1

**Fig. 5:**
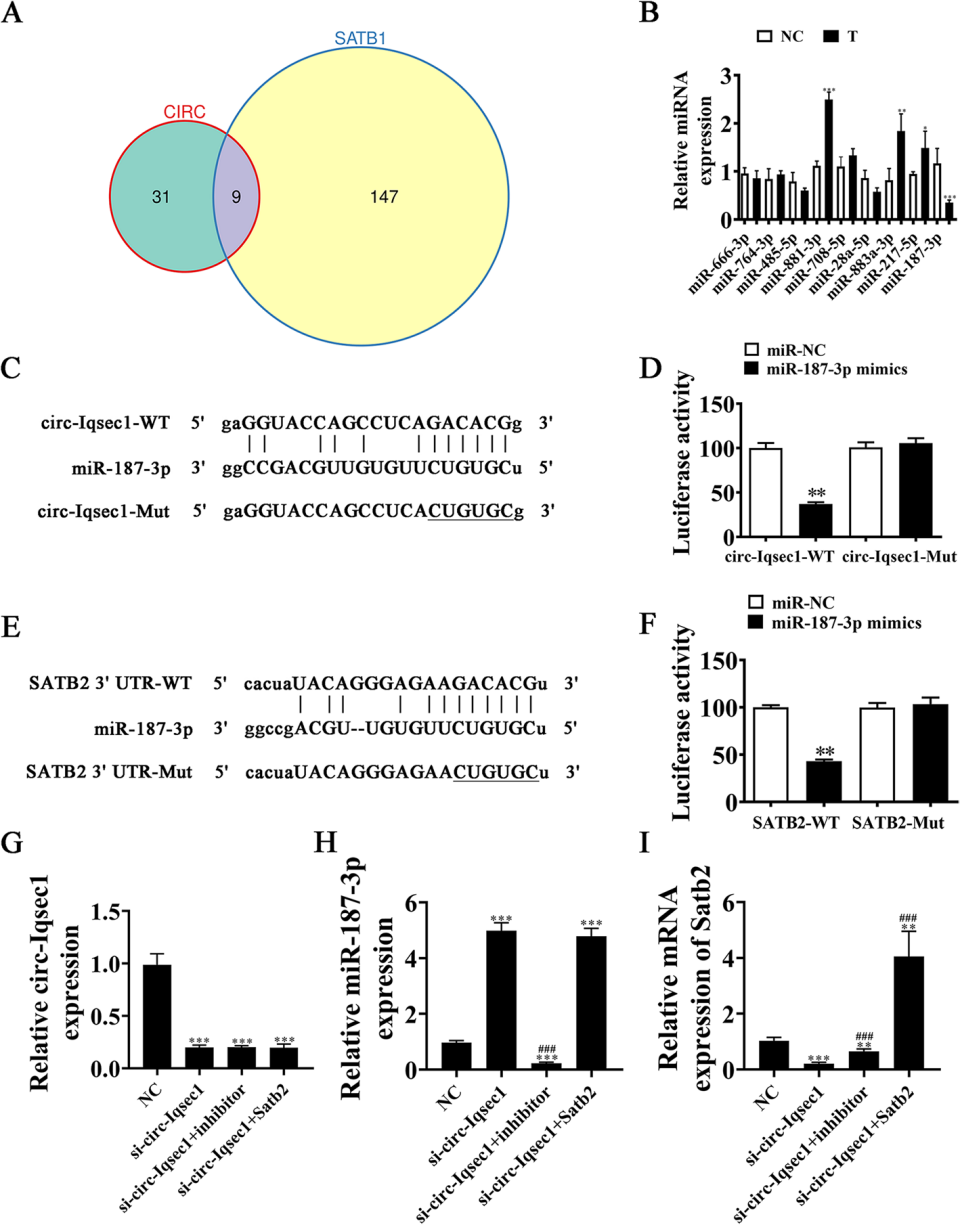
The miR-187-3p links *Satb2* and circ-*Iqsec1*. **A** Venn diagram showing that miRNA interacts with both Satb2 and circ-Iqsec1. **B** RT-qPCR data giving nine miRNA expressions in BMSCs post osteogenic induction for 0 (NC) and 3 (T) weeks. **C** Bioinformatics analyses predicting miR-187-3p binding sites in circ-Iqsec1. MUT version regarding circ-Iqsec1 is provided. **D** Relative luciferase activity 2 d post HEK293T cell transfection with miR-187-3p mimic/NC or circ-Iqsec1 WT/MUT. **E** miR-187-3p binding site predictions regarding *Satb2* 3'-UTR. Mutant 3′-UTR-*Satb2* version is given. **F** Relative luciferase activity 2 days post-HEK293T cell transfection through miR-187-3p mimic/NC or 3′-UTR-*Satb2* WT/Mut. **G**–**I** RT-qPCR results giving circ-Iqsec1, miR-187-3p, and *Satb2* expressions in BMSCs after transfection with si-circ-*Iqsec1*, miR-187-3p inhibitor, or *Satb2* overexpression vector alone or in combination. Outputs are expressed as the mean ± SD. ^*^P < 0.05, ^**^P < 0.01, ^***^P < 0.001 vs. NC. ^###^*P* < 0.001 vs. si-circ-Iqsec1

Studies have found that miRNA mediates circRNA and mRNA regulation [17, 18]. Target miRNAs connecting *Satb2* and circ-*Iqsec1* were sought. Bioinformatics analyses found that circ-*Iqsec1* interacts with many miRNAs such as miR-1224-5p, miR-666-3p, miR-3068-5p, miR-3075-5p, miR-764-3p, miR-132-5p, miR-187-3p, miR-485-5p, miR-3081-3p, miR-3076-3p, miR-3110-5p, miR-674-5p, and miR-3473d. Further bioinformatics analysis found that the 3′-UTR of Satb2 could interact with miR-15b-5p, miR-23b-3p, miR-101a-3p, miR-124-3p, miR-128-3p, miR-132-3p, miR-140-3p, miR-144-3p, miR-153-3p, and miR-187-3p. Venn diagram analysis showed that only nine miRNAs could interact with both 3′-UTR-*Satb2* and circ-Iqsec1 (Fig. 5A). RT-qPCR detection showed that miR-187-3p expression decreased significantly in BMSCs in the osteogenic differentiation-induced group (Fig. 5B).

Luciferase reporter outcomes validated that miR-187-3p inhibited luciferase activity in WT but not MUT cells (Fig. 5C–D), showing that miR-187-3p was a circ-*Iqsec1* target.

Data showed that *Satb2* was an miR-187-3p downstream target. MUT or WT 3′-UTR-*Satb2* sequences, such as the miR-187-3p binding sequence, were transfected into a luciferase reporter vector to validate correlations between *Satb2* and miR-187-3p (Fig. 5E). A luciferase reporter vector was transfected into HEK293 cells with or without miR-187-3p mimic. The luciferase reporter outputs showed that miR-187-3p suppressed luciferase activity in WT but not MUT cells (Fig. 5F), implying that *Satb2* was an miR-187-3p target.

RT-qPCR data showed that circ-Iqsec1 expression decreased post-transfection with si-circ-*Iqsec1*. However, treatment with miR-187-3p inhibitor or *Satb2* overexpression vector (*Satb2*) did not restore circ-*Iqsec1* expression in BMSCs (Fig. 5G). This finding suggested that *Satb2* and miR-187-3p were circ-Iqsec1 downstream targets. RT-qPCR results revealed that circ-*Iqsec1* silencing increased miR-187-3p expression. *Satb2* overexpression did not reverse si-circ-Iqsec1-induced miR-187-3p upregulation (Fig. 5H), suggesting that miR-187-3p was located downstream of circ-*Iqsec1*. The result also showed that circ-Iqsec1 silencing decreased *Satb2* expression. miR-187-3p downregulation restored *Satb2* expression after si-circ-Iqsec1. After transfection with *Satb2* overexpression vector, *Satb2* expression increased significantly (Fig. 5I). This suggested that circ-*Iqsec1* enhanced *Satb2* expression via sponging miR-187-3p.

### miR-187-3p downregulation or Satb2 overexpression restores BMSC osteogenic differentiation ability after silencing circ-Iqsec1

**Fig. 6:**
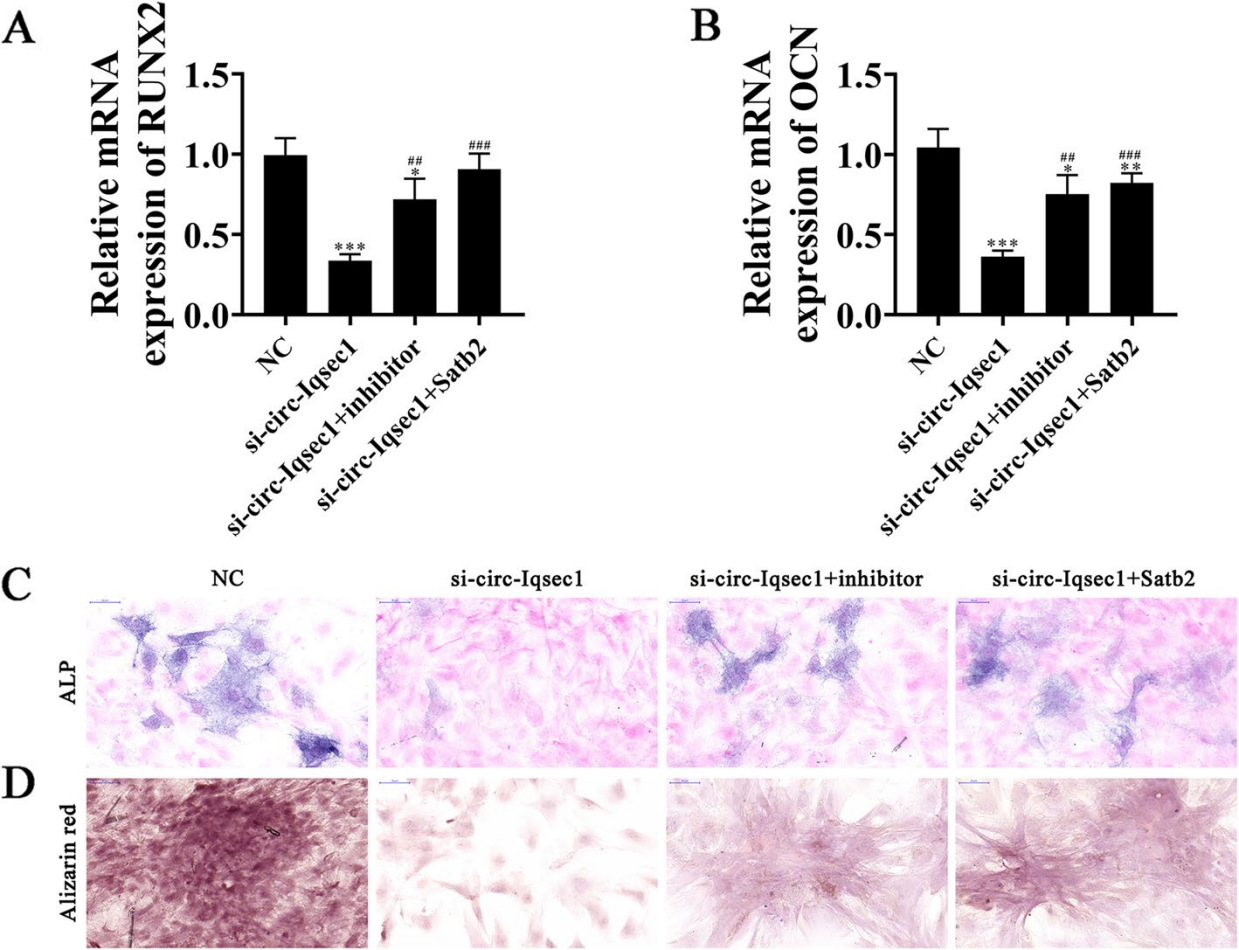
miR-187-3p downregulation or *Satb2* overexpression restored BMSC osteogenic differentiation ability after silencing circ-*Iqsec1*. **A**, **B** RT-qPCR data showing *RUNX2* (**A**) and *OCN* (**B**) expression. Results are expressed as the mean ± SD. ^*^P < 0.05, ^**^P < 0.01, ^***^P < 0.001 vs. NC. ^##^*P* < 0.01, ^###^*P* < 0.001 vs. si-circ-Iqsec1. **C**, **D** Immunohistochemical staining for *ALP* and alizarin red staining for calcium showing BMSC osteogenic differentiation ability

RT-qPCR detection showed that *RUNX2* and *OCN* expressions decreased post-circ-*Iqsec1* silencing. While miR-187-3p downregulation or Satb2 overexpression restored both *RUNX2* and *OCN* expression (Fig. 6A, B), immunohistochemical staining for *ALP* and alizarin red staining for calcium showed that miR-187-3p downregulation or *Satb2* overexpression restored osteogenic differentiation post-circ-Iqsec1 silencing in BMSCs.

### Satb2 upregulation restores BMSC osteogenic differentiation ability post-miR-187-3p overexpression

**Fig. 7:**
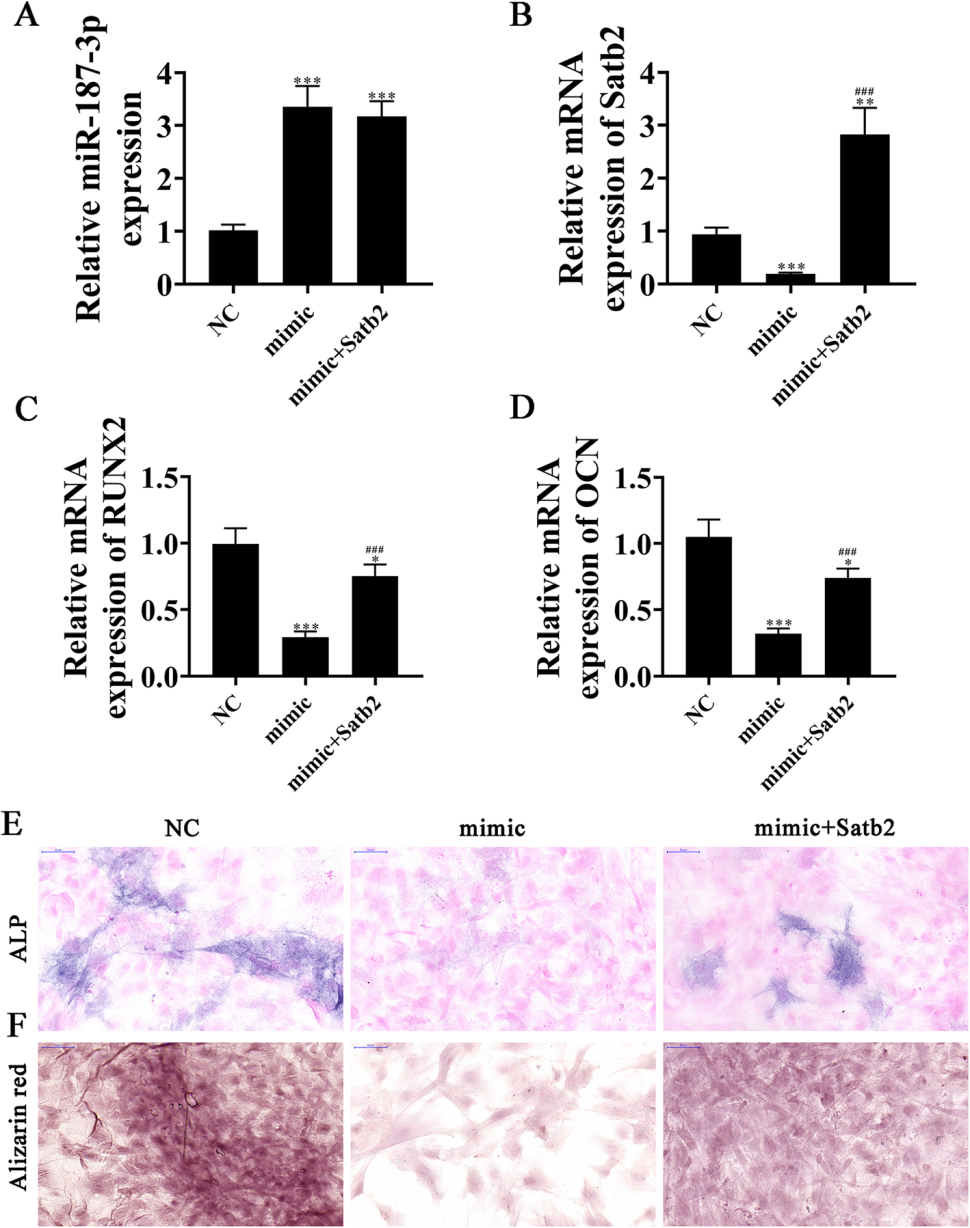
Upregulation of *Satb2* restored the osteogenic differentiation ability of BMSCs after overexpression of miR-187-3p. **A**, **B** RT-qPCR detection shows the expression of miR-187-3p and *Satb2*. Data are expressed as the mean ± SD. ^**^P < 0.01, ^***^P < 0.001 vs NC. ^###^*P* < 0.001 vs mimic. **C**, **D** RT-qPCR detection shows the expression of *RUNX2* (**A**) and *OCN* (**B**). Data are expressed as the mean ± SD. ^*^P < 0.05, ^***^P < 0.001 vs NC^###^*P* < 0.001 vs mimic. **E**, **F** Immunohistochemical staining for *ALP* and alizarin red staining show the osteogenic differentiation ability of BMSCs

RT-qPCR detection showed that miR-187-3p expression increased in BMSCs post-miR-187-3p mimic transfection. However, overexpression of *Satb2* could not reverse miR-187-3p expression after miR-187-3p mimic transfection (Fig. 7A). RT-qPCR data showed that *Satb2* expression decreased post-miR-187-3p mimic transfection. However, after transfection with *Satb2*, *Satb2* expression significantly increased (Fig. 7B). RT-qPCR detection showed that *RUNX2* and *OCN* expressions decreased post-miR-187-3p overexpression. However, overexpression of Satb2 restored both *RUNX2* and *OCN* expression (Figs. 7C, 7D). Immunohistochemical staining for *ALP* and alizarin red staining for calcium illustrated that *Satb2* overexpression restored osteogenic differentiation post-miR-187-3p upregulation in BMSCs (Fig. 7E, F).

### miR-187-3p downregulation or Satb2 overexpression restores BMSC osteogenic differentiation ability after silencing circ-Iqsec1 in vivo

**Fig. 8:**
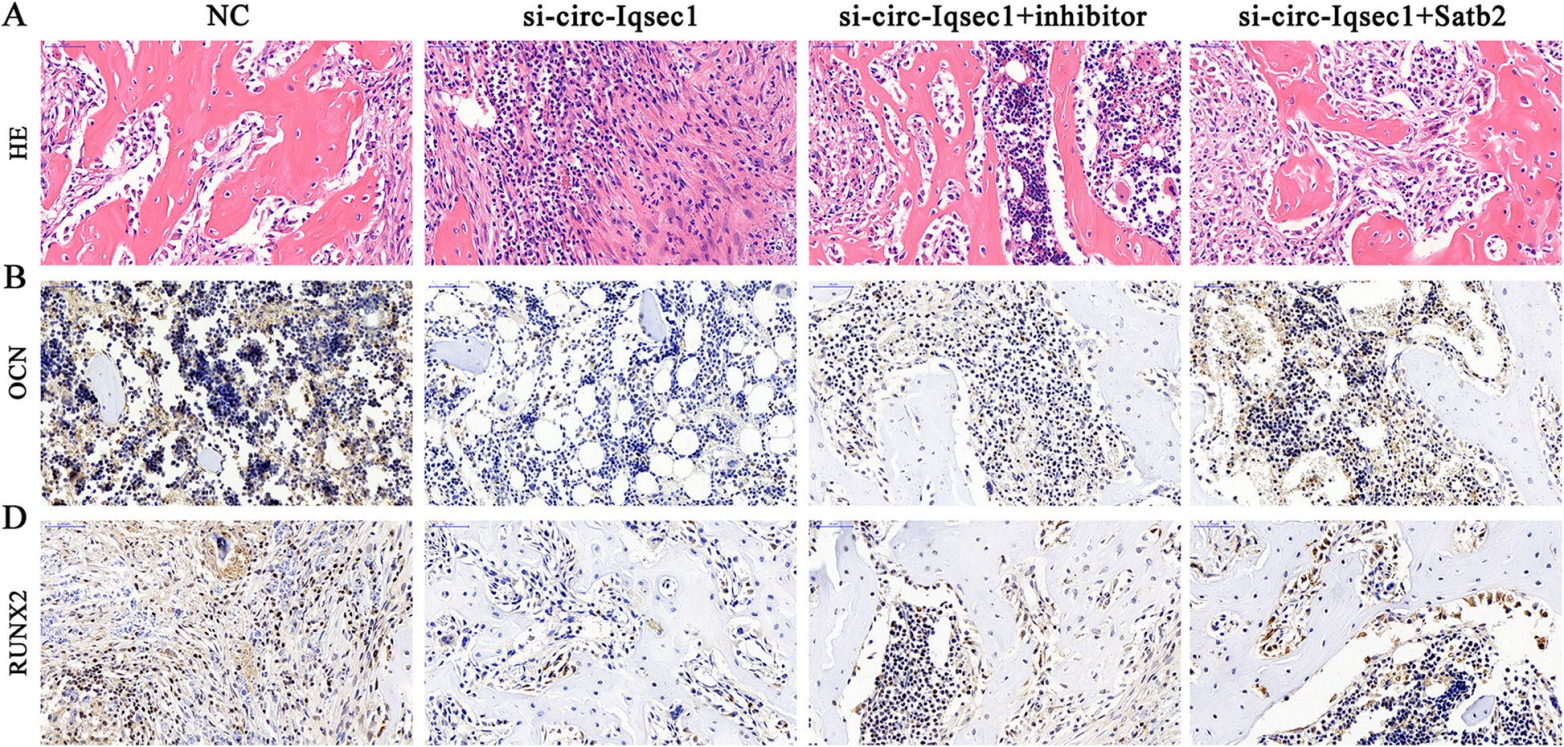
Downregulation of miR-187-3p or overexpression of *Satb2* restored the osteogenic differentiation ability of BMSCs after silencing circ-*Iqsec1 in vitro*. **A**–**C** immunohistochemical staining with HE, and *OCN* and *RUNX2* staining show the changes in tissue structure and protein expression

To investigate whether circ-*Iqsec1* enhances bone formation in the mouse model, BMSCs transfected with si-circ-*Iqsec1*, miR-187-3p inhibitor, or *Satb2* overexpression vectors were loaded onto scaffolds that were implanted in the subcutaneous space in nude mice. After 8 weeks, we harvested the implantation samples, which we embedded in paraffin, sectioned, deparaffinized, and stained with H&E or for *OCN* and *RUNX2*. H&E staining showed little bone formation in the si-circ-Iqsec1 group, whereas osteoid formation was restored after miR-187-3p downregulation or *Satb2* overexpression (Fig. 8A). Immunohistochemical staining for *OCN* and *RUNX2* showed that miR-187-3p downregulation or *Satb2* overexpression restored both *OCN* (Fig. 8B) and *RUNX2* (Fig. 8C) expression after silencing circ-*Iqsec1*.

## Discussion

OP is a well-defined trait that leads to increased mortality and morbidity [18]. Because of the pluralistic differentiation potential, BMSCs function to regulate the microenvironment and bone mass and strength [19, 20]. The present investigation discovered that BMSCs have osteogenic differentiation potential. In addition, circRNA was reported to function importantly in osteogenic differentiation [21]. High-throughput sequencing elucidated that circRNA had abnormal expression in BMSCs during osteogenic differentiation and that circ-*Iqsec1* expression increased in BMSCs during osteogenic differentiation. circ-Iqsec1 downregulation inhibited *RUNX2* and *OCN* expression and BMSC osteogenic differentiation. The data suggested that circ-Iqsec1 functioned in BMSC osteogenic differentiation.

High-throughput sequencing for mRNA expression detection showed abnormal mRNA expression in BMSCs regarding osteogenic differentiation. Special AT-rich sequence-binding protein 2 (*Satb2*) expression was increased in BMSCs during osteogenic differentiation. Enhanced SATB2 has been reported to promote osteogenic differentiation of BMSCs from patients with osteonecrosis induced by ethanol [22, 23]. Previous studies have found that *Satb2* was a particular immunohistochemical biomarker of osteoblastic differentiation and has been helpful regarding bone and soft tissue tumors [24, 25]. *Satb2* downregulation inhibited *RUNX2* and *OCN* expression and BMSC osteogenic differentiation. This is consistent with former investigations showing that Satb2 functions in BMSC osteogenic differentiation.

Previous studies have shown that circRNAs might regulate gene expression by influencing transcription, mRNA turnover, and translation via sponging RNA-binding proteins and microRNAs [18, 26, 27]. This study aimed to identify the connecting target miRNA for *Satb2* and circ-*Iqsec1*. Bioinformatics analysis and luciferase reporter analysis confirmed that miR-187-3p functioned to link circ-Iqsec1 and Satb2. Former investigations showed that miR-187-3p expression decreased during osteogenic differentiation of human adipose-derived mesenchymal stem cells [28]. miR-187-3p expression upregulation inhibited the osteogenic differentiation of osteoblast precursor cells by inhibiting cannabinoid receptor type 2 [29]. The present investigation verified that miR-187-3p expression decreased in BMSCs during osteogenic differentiation.

The data discovered that miR-187-3p downregulation or *Satb2* overexpression restored the osteogenic differentiation capability of BMSCs post-silencing circ-*Iqsec1* in in vivo and in vitro investigations. *Satb2* upregulation restored BMSC osteogenic differentiation capability post-miR-187-3p overexpression.

## Conclusion

The present study revealed that circ-*Iqsec1* functioned during osteogenic differentiation of BMSCs. circ-*Iqsec1* induced BMSC osteogenic differentiation by regulating the miR-187-3p/*Satb2/RUNX2/OCN* signaling pathway. In addition, the effect of circ-*Iqsec1* on osteogenic differentiation may be explored in the near future.

## Data Availability

The datasets used and/or analyzed during the current study are available from the corresponding author on reasonable request.
